# Treatment outcome and survival status among adult patients treated for lupus nephritis in selected tertiary hospitals of Ethiopia

**DOI:** 10.1038/s41598-024-56317-6

**Published:** 2024-03-07

**Authors:** Oumer Aliyi, Berhanu Worku, Minimize Hassen, Oumer Sada Muhammed

**Affiliations:** 1 Department of Pharmacy, College of Medicine and Health Sciences, Madda Walabu University, Bale Goba, Ethiopia; 2 Department of Internal Medicine, School of Medicine, College of Health Sciences, St. Paul’s Hospital Millennium Medical College, Addis Ababa, Ethiopia; 3 Department of Clinical Pharmacy, School of Pharmacy, College of Medicine and Health Sciences, Wollo University, Dessie, Ethiopia; 4 Department of Pharmacology and Clinical Pharmacy, School of Pharmacy, College of Health Sciences, Addis Ababa University, Addis Ababa, Ethiopia

**Keywords:** Health care, Medical research, Nephrology

## Abstract

Lupus nephritis (LN) is kidney involvement of systematic lupus erythematous that ranges from mild to severe and occurs in 60% of adult patients. Despite advances in therapy, LN morbidity and mortality remains high. There is a paucity of data regarding adult LN patient's treatment outcome, survival status, and associated factors in developing countries, particularly in Ethiopia. This study aimed to assess the treatment outcome, survival status, and associated factors of adult patients treated for LN in two selected tertiary hospitals [Tikur Anbessa Specialized Hospital (TASH) and St. Paul’s Hospital Millennium Medical College (SPHMMC)] of Addis Ababa, Ethiopia. A hospital-based retrospective cross-sectional multicenter study was conducted from January 1, 2016 to January 1, 2021. Socio-demographic, clinical, and treatment-related data were collected from patient’s medical records by using a structured abstraction checklist. Descriptive statistics were used to summarize the quantitative data as appropriate. The modified Aspreva Lupus Management Study (mALMS) criteria was applied to categorize LN treatment outcomes into complete, partial, and non-response. Multinomial logistic regression analysis was performed to identify predictors of LN treatment outcome. Patients’ survival was estimated by using Kaplan–Meier and Cox proportion regression analysis. *P* value < 0.05 was considered to declare statistical significance. A total of 200 LN patients were included in the final analysis. Amongst these, the majority of them (91.5%) were females. The median age of the patients was 28 (15–60) years. The mean duration of treatment follow-up was 28 months. The commonly prescribed immunosuppressive drugs during both the induction (49.5%) and maintenance (60%) phases were a combination of mycophenolate mofetil with prednisolone. Complete, partial, and non-responses at the last follow-up visit accounted for 66.5%, 18.0%, and 15.5%, respectively. Patient survival at the last follow-up visit was more than 90% for patients with complete response to the induction therapy. Non-response at the last follow-up visit was significantly associated with severe disease activity index (adjusted odds ratio [AOR] = 6.25, 95% confidence interval [CI] 1.49–26.10), presence of comorbidity (AOR = 0.21, 95% CI 0.05–0.92), baseline leucopenia (AOR = 14.2, 95% CI 1.04–201.3), partial response at the end of induction therapy (AOR = 32.63, 95% CI 1.4–736.0), and duration of induction therapy of greater than 6 months (AOR = 19.47, 95% CI 1.5–258.8). This study unveiled that lower numbers of LN patients were presented with non-response at the last follow-up visit and non-response to induction therapy was associated with lower patients’ survival rates compared with complete or partial response.

## Introduction

Lupus nephritis (LN) is the most common complication of systemic lupus erythematosus (SLE) which is caused by the complex interaction between genetic predisposition and environmental factors such as excess sunlight exposures, infection, extreme stress, and certain drugs^1^. It is estimated that lupus nephritis clinically affects around 60% of SLE patients^2^. The prevalence of LN remained unchanged over the last 45 years, with 50%, 25%, and 20% accounting for class III, IV, and V, respectively^2^. LN is manifested by an increase in serum creatinine, development of proteinuria (> 0.5 g/day), or active urinary sediment with red blood cells, granular or mixed cast that results in end-stage renal disease (ESRD).

Based on renal biopsy findings, LN was classified into class I (minimal mesangial involvement), class II (mesangial proliferative LN), class III (focal LN involving < 50% of glomeruli), class IV (diffuse segmental LN involving > 50% of glomeruli), class V (lupus membranous nephropathy), and class VI (advanced stage affecting > 90% of glomeruli) as per the International Society of Nephrology (ISN) and the Renal Pathology Society (RPS)^3,4^.

Treatment of LN is not an easy task. Despite all the studies that have been conducted and the use of various advanced drugs, treating LN poses a significant challenge. Although different protocols were used depending on the histological class of LN, the exact standard treatment regimen for LN patients remains controversial. Class II LN patients often have excellent renal prognosis by prednisolone only. Unless there is an extra-renal manifestation, class I usually does not require specific immunosuppressive therapy^5^. However, if not treated well, class II LN can transform into class III or IV^6,7^. For class VI, renal transplantation is preferred over immunosuppressive therapy^7,8^. Among all the treated patients, 10–15% progress to ESRD. Even with treatment, up to 44% of patients with class III or IV LN develop ESRD within 15 years^6,7^.

To achieve the desired treatment outcome, LN treatment is divided into two phases: induction therapy and maintenance therapy. Induction therapy consists of high-dose immunosuppressant drugs used for six months to decrease disease activity, whereas maintenance therapy consists of less intensive immunosuppressant drugs used to maintain remission and prevent disease relapse in patients who respond to induction therapy^9^. According to the recent American College of Rheumatology (ACR) guideline, patients with biopsy-proven LN class III or IV should receive induction therapy of either mycophenolate mofetil (MMF) or intravenous cyclophosphamide along with high-dose corticosteroid^4^. The duration of the maintenance phase is still arguable^5,9^.

The definition of categorizing LN response into complete response (CR), partial response (PR), and non-response (NR) to drug therapy varies from study to study. The modified Aspreva Lupus Management Study (mALMS) criteria, which classifies LN response into complete, partial, and no response by considering serum creatinine and 24-h urine protein, was widely used in most studies^10^. The response to therapy in LN patients is affected by numerous factors including patient, clinical and treatment-related factors. In Africa, the response is limited by the availability and cost of drugs, and by the shortage of laboratory facilities and poor drug adherence^11,12^.

According to internationally accepted guidelines, the last CR is often expected after 24 months of therapy, despite reports of CR at 6 months, 12 months, and 36 months in a few studies^13^. These realities differ based on ethnicity, baseline clinical characteristics, regimen chosen, and initial Standard Systemic Lupus Erythematous Disease Activity Index (SLEDAI-2 K) score^14^. When combined with MMF, adjuvant therapy with hydroxychloroquine is used to increase CR^15^. A nascent study asserted that CR varies among studies and may range from 10 to 85%^16^.

Although some studies have been conducted in developed countries on the treatment outcome, and survival status of LN, the issue is under-studied in sub-Saharan Africa, particularly Ethiopia. In most of these studies, complete response [CR] and partial response [PR] were merged and considered similar outcomes, despite being clinically distinct. In Ethiopia, there is a knowledge gap about factors associated with non-response to therapy and the survival status of LN patients. So, the current study was designed to fill these gaps of knowledge. This study aimed to assess the treatment outcome, survival status, and associated factors among adult patients treated for LN in TASH and SPHMMC from January 1, 2016 to January 1, 2021.

## Methodology

### Study area, study setting, and study period

A hospital-based retrospective cross-sectional study was conducted at the adult outpatient renal clinics of TASH and SPHMMC, Addis Ababa, Ethiopia from January 1, 2016-January 1, 2021. Data was collected from July 1-September 1, 2021. TASH is the largest tertiary care, specialized, referral, and teaching hospital in the country that is owned by the government and was established in 1973. It has 51 specialty outpatient clinics, serving 500,000 patients annually^17^. SPHMMC is a major teaching hospital inaugurated in 1969 by Emperor Haile Selassie with the help of the German Evangelical Church and currently has more than 700 beds, with an annual average of 200,000 patients served in the hospital^18^. The outpatient renal clinics of both hospitals offer comprehensive clinical and follow-up services in which adult patients with renal diseases including LN were followed. Renal biopsy was provided in both hospitals.

### Study participants

All patients who fulfilled the inclusion criteria were included in the study. All adult patients with LN, who were on follow-up at either TASH or SPHMMC renal clinic between January 2016 and January 2021 were included in the study. Patients were eligible for enrollment in this study if they were at least 15 years of age at diagnosis, diagnosed with LN either by renal biopsy or laboratory results of 24 h urine protein and lupus serology, had SLE based on the 1982 revised ACR criteria, who have had regular follow-up at the adult renal clinic of either TASH or SPHMMC for at least 12 months, had sufficient laboratory data for assessment of renal remission after 6 month, and on standard treatment protocol. Patients with ESRD before starting treatment, had Class I and VI types of LN, with known chronic kidney disease and diabetes prior to LN onset, had double glomerulonephropathy, and with incomplete medical records were excluded.

### Sample size determination and sampling technique

All adult LN patients who met the inclusion criteria and had follow-ups in TASH and SPHMMC were included as study participants since a limited number of patients were encountered during the study period. A convenience sampling technique was used to collect the necessary data that fulfils the inclusion criteria. The two health facilities were chosen for convenience because they had a large number of LN patients and were providing renal biopsies for LN patients.

### Study variables

#### Dependent variable

Treatment outcome status (complete response at the last follow-up [CR], partial response at the last follow-up [PR], non-response at the last follow-up [NR]).

#### Independent variables

(1) Sociodemographic characteristics include age, sex, and place of residence. (2) Clinical characteristics include SLE duration before LN onset, class of LN, SLE disease activity, presence of comorbidity, leucopenia, thrombocytopenia or anemia, baseline serum creatinine, and 24 h urine protein, and baseline ANA and anti-DsDNA. (3) Treatment-related characteristics include treatment regimen selected, pulse steroid therapy, response to induction therapy, time to remission, treatment duration, total follow up period, admission during follow up, and cotrimoxazole infection prophylaxis.

### Data collection instrument and procedure

Data was collected by using a structured checklist. The checklist was developed after reviewing different literature published on the subject area. It contained necessary variables that could be obtained from the patient’s medical profile, like patient-related data (age, sex, diagnosis, disease duration, baseline clinical information, laboratory data, and disease severity score by SLEDAI-2K, medication-related data (regimen selected, dose, and duration) and outcome status (CR, PR, and NR). The SLEDAI-2K score was used to assess the SLE disease severity index^19^. Data were retrospectively collected from the patient’s medical record by strictly following criteria needed to confirm LN like renal biopsy. In the absence of renal biopsy, the presence of two consecutive 24-h proteinuria readings > 0.5 g/day, and an additional feature supporting active lupus, such as positive serology or active urinary sediment was considered to diagnose LN^20^. A pre-test was done on 5% of the sample population to assure clarity and content uniformity. The checklist was amended based on the pretest finding. Two days training was given by the principal investigators for two clinical pharmacists (data collectors) about the aim of the study, the checklist, and data collection procedures. Initially, SLE patients with LN were counted from the patient registration logbook. Then, by using patient’s identification card number, patient’s medical chart, and I-care profile, data were retrieved from the card room and I-care respectively. However, as this study was retrospective, adverse drug events were not collected unless recorded by the physicians.

### Data analysis

The data were entered into and cleaned in Epi Info version 4.6.0.2 and were exported into and analyzed in Statistical Package for the Social Sciences (SPSS) version 26. Initially, normality and multicollinearity were checked. A normality test was done by using Shapiro–Wilk’s and Kolmogorov-Simonov (K-S) tests for numerical independent variables. To declare the absence of normality, a level of significance greater than 0.05 was used. Multicollinearity was checked to test the correlation among predictor variables using the variance inflation factor (VIF). A VIF < 8 was considered a cut point for excluding collinearity. Frequencies and percentages were used for categorical variables, while mean ± standard deviation and/or median (IQR) for continuous variables.

A multinomial logistic regression analysis was carried out to analyze the association between independent variables and treatment outcomes. The maximum likelihood of parameter estimators for variables composing the model was used to obtain crude odds ratio (COR) and adjusted odds ratio (AOR) with a 95% confidence interval. The validity of the model was tested by likelihood ratio test. During multinomial analysis, the LN treatment outcome category labeled as NR was considered as the reference category against which all other outcomes were compared. Different reference categories were used when comparing variables categorized within the same variable. All variables associated with LN treatment outcome at *p*-value ≤ 0.25 in the bivariate analysis were picked and entered into the multivariate analysis to control confounders.

Survival analysis (Kaplan–Meier method) was used to calculate the survival rate by total follow-up time between variables. The overall survival curves were derived by Kaplan–Meier methods and the difference between the survival curves were compared by using a log-rank test. The Cox regression model assumption of proportional hazards was checked by testing the interaction of covariates with time before running the Cox proportional hazard regression analysis using Cox with time-dependent covariates. Multivariate Cox proportional hazard regression analysis was performed to identify independent factors associated with non-response (NR). For measuring the strength of association, the hazard ratio (HR) was used. The level of statistical significance was declared at *p*-value < 0.05 and results were reported at a 95% confidence interval.

### Operational definition

Complete response (CR) at the last follow-up—is reducing serum creatinine to ≤ 1.4 mg/dl and 24-h urine protein to ≤ 0.5 g/day. Partial response (PR) at the last follow-up—is occurrence of either serum creatinine to ≤ 1.4 mg/dl or 24-h urine protein ≤ 0.5 g/day. Non-response (NR) at the last follow-up—is failure to achieve complete [CR] or partial response [PR]), death, development of ESRD, and relapse. Relapse—is nephrotic range proteinuria (> 3 g/24 h), active urinary sediment, and an increase in serum creatinine by 30% after achieving complete or partial remission. End-stage renal disease (ESRD)-serum creatinine > 6 mg/dl at the last follow-up visit, on renal dialysis for at least 3 months, and waiting for renal transplantation. Comorbidity—is the presence of cardiovascular disease, neurologic disease, dyslipidemia, cancer, infection, hypothyroidism, psychosis, and drug-induced complications. Baseline clinical characteristics—it is the patient clinical characteristics and laboratory data at the first diagnosis of lupus nephritis as registered on the patient's medical card. Baseline WBC—is the reading of WBC at the initial diagnosis of lupus nephritis before starting treatment of lupus nephritis as registered on the patient’s medical record. Mild disease activity—Lupus nephritis patients with SLEDAI-2 K score between 1 and 3 inclusive. Moderate disease activity—Lupus nephritis patients with SLEDAI-2 K score between 4 and 12 inclusive. Severe disease activity—Lupus nephritis patients with SLEDAI-2 K score above 12.

### Ethical consideration

The study was approved by the Ethical Review Board (ERB) of Addis Ababa University, College of Health Sciences (25/03/2021; ERB No. 252/13/2021), and the Institutional Review Board of SPHMMC (Reference number; Pm23/384). The study protocol was performed in accordance with the Declaration of Helsinki. All methods were performed in accordance with the relevant guidelines and regulations. informed consent was obtained from all subjects. To collect the necessary data, official letters were written to each hospital and permission to conduct the study was obtained from responsible directorates of each hospital. Confidentiality, neutrality, anonymity, accountability, and academic honesty were maintained throughout the study.

## Results

### Socio-demographic and clinical characteristics of lupus nephritis patients

A total of 216 LN patients were enrolled from TASH and SPHMMC. Of these, 16 patients were lost from follow-up, and thus only 200 patients were included in the final analysis. Out of the total studied participants, 183 (91.5%) were females. The median (IQR) age of patients was 28 (33–23) years. The median duration of SLE before clinical diagnosis of LN onset was 9.5 month and 98% of the patients were considered early-onset LN. The mean duration of observation in LN patients included in our study during the study periods was 28 months. Renal involvement of LN was verified by renal biopsy in only 73.5% of patients, possibly due to the patient's financial constraints in the study setting. Mean baseline serum creatinine and 24-h urine protein were 2.04 ± 1.75 mg/dl, and 2.4 ± 0.52 g/day, respectively. About 79(39.5%) of patients had a baseline serum creatinine of ≤ 1.4 mg/dl and 80(40%) of patients had a baseline 24-h urine protein of > 3 g/day. Approximately 38.5% of patients had a variety of comorbidities (Table 1).

**Table 1:** Baseline socio-demographic and clinical characteristics of patients with LN according to treatment outcome in TASH and SPHMMC from January 1, 2016 to January 1, 2021 (n = 200).

Variables	Category	All patient, n	Treatment outcome			*P* value
			CR, n (%)	PR, n (%)	NR, n (%)	
Age	15–30 years	127	81 (40.5)	27 (13.5)	19 (9.5)	0.140
	> 30 years	73	52 (26)	9 (4.5)	12 (6)	
Sex	Female	183	124 (62)	31 (15.5)	28 (14)	0.384
	Male	17	9 (4.5)	5 (2.5)	3 (1.5)	
LN diagnosis	No biopsy	53	32 (16)	10 (5)	11 (5.5)	0.787
	With biopsy	147	96 (48)	31 (15.5)	20 (10)	
Residence	Outside AA^a^	107	74 (37)	20 (16)	13 (6.5)	0.579
	From AA	93	59 (29.5)	16 (8)	18 (9)	
SLE duration	≤ 5 years	196	131 (65.5)	35 (17.5)	30 (15)	0.650
	> 5 years	4	2 (1)	1 (0.5)	1 (0.5)	
Class of LN	Class II	23	13 (6.5)	10 (5)	0 (0)	0.121
	Class III	43	29 (14.5)	7 (3.5)	7 (3.5)	
	Class IV	59	39 (14.5)	11 (5.5)	9 (4.5)	
	Class V	9	6 (3)	1 (0.5)	2 (1)	
	Mixed	13	9 (4.5)	2 (1)	2 (1)	
	Unknown	53	32 (16)	10 (5)	11 (5.5)	
SLEDAI-2 K	Moderate	153	107 (65.5)	27 (13.5)	19 (9.5)	0.203
	Severe	57	36 (13)	9 (4.5)	12 (6)	
Comorbidity	Yes	77	50 (25)	10 (5)	17 (8.5)	0.147
	No	123	83 (41.5)	26 (13)	14 (7)	
Baseline SCr (mg/dl)	≤ 1.4 mg/dl	79	83 (41.5)	26 (13)	14 (7)	0.176
	> 1.4 mg/dl	121	77 (38.5)	22 (11)	22 (11)	
Baseline uPCR	≤ 3 g/day	120	88 (44)	14 (7)	18 (9)	0.012
	> 3 g/day	80	45 (22.5)	22 (11)	13 (6.5)	
ANA	Positive	167	111 (55.5)	30 (15)	26 (13)	0.489
	Negative	29	20 (10)	4 (2)	5 (2.5)	
	Unknown	4	2 (1)	2 (1)	0 (0)	
Anti-dsDNA	Positive	76	50 (25)	14 (7)	12 (6)	0.601
	Negative	20	15 (7.5)	1 (0.5)	4 (2)	
	Unknown	104	68 (34)	21 (10.5)	15 (7.5)	
Baseline Hgb	≤ 10 g/dl	87	54 (27)	19 (15.7)	14 (13.5)	0.417
	> 10 g/dl	113	79 (39.5)	17 (8.5)	17 (8.5)	
Baseline PLT	≤ 150 × 10^9^/L	35	25 (12.5)	5 (2.5)	5 (2.5)	0.771
	> 150 × 10^9^/L	165	108 (54)	31 (15.5)	26 (13)	
Baseline WBC	≤ 3 × 10^9^/L	39	33 (16.5)	5 (2.50)	1 (0.5)	0.015
	> 3 × 10^9^/L	161	100 (50)	31 (15.5)	30 (15)	
Admission	Yes	73	40 (20)	14 (7)	19 (9.5)	0.005
	No	127	93 (46.5)	22 (11)	12 (6)	
Infection	Yes	51	26 (130)	14 (7)	11 (5.5)	0.023
	No	149	107 (53.5)	22 (11)	20 (10)	

*LN* lupus nephritis, *SLE* systemic lupus erythematous, *AA* addis ababa, *Hgb* hemoglobin, *PLT* platelet, *WBC* white blood cell, *SCr* serum creatinine, *uPCR* urine protein creatinine ratio, *ANA* antinuclear antibodies, *dsDNA* double strand DNA, *Outside AA*^*a*^ Oromia, Amhara, Southern Nation and Nationality People, Tigray, Somali, and Harari region, *CR* complete response, *PR* partial response, *NR* non-response, *SLEDAI-2 K* systemematic lupus erythematous disease activity index 2002.

### Treatment outcomes and causes of admission of lupus nephritis patients

About 36.5% of LN patients had a history of admission to a hospital during their follow-up time. The most common causes of admissions were acute kidney injury (11.5%), and pneumonia (9%). Overall remission (complete plus partial response) was achieved in 84.5% of patients and non-response to drug therapy occurred in 15.5% of patients at their last follow-up visit (Table 2).

**Table 2:** Prevalent causes of admission and treatment outcomes of patients with LN on follow-up in the renal clinic of TASH and SPHMMC from January 1, 2016 to January 1, 2021 (n = 200).

	Category	N	%
Causes of admission	Acute kidney injury	23	11.5
	Pneumonia	18	9.0
	Pulmonary tuberculosis	7	3.5
	Deep vein thrombosis	3	1.5
	Anemia	5	2.5
	Abortion	1	0.5
	Anasarca	3	0.5
	Sepsis	3	0.5
	Delivery	2	1.0
	Relapse	3	1.5
	Seizure	2	1.0
	Immune thrombocytopenia	1	0.5
	Peptic ulcer disease	1	0.5
	Hypertensive emergency	1	0.5
LN treatment outcome	Complete response [CR]	133	66.5
	Partial response [PR]	36	18.0
	Non-response [NR]	31	15.5

### Types of comorbidities of lupus nephritis patients

Hypertension was the most prevalent comorbid condition manifested in LN patients, constituting 22.5% of all comorbidities. Amlodipine (9.5%) and Enalapril (9.5%) were the most frequent drugs used to manage comorbidities. The most common immunosuppressant drugs that cause comorbidity were prednisolone and chloroquine, as recorded by physicians (Table 3).

**Table 3:** Types of comorbidities and drug-induced complications in patients with LN on follow-up in the renal clinic of TASH and SPHMMC from January 1, 2016 to January 1, 2021 (n = 200).

Type of comorbidity	Total	Percent	Drug-induced comorbidity	Total	Percent
Hypertension	45	22.5	Maculopathy	2	1.0
Short sight	2	1	Ophthalmopathy	2	1.0
Raynaud phenomenon	2	1	Osteonecrosis	1	0.5
Deep vein thrombosis	4	2	Cushing’s syndrome	2	1.0
Hypertension + DVT	3	1.50	Cataract	1	0.5
Hypothyroidism	3	1.50	Short sight	2	1.0
Maculopathy	2	1	Steroid-induced myopathy	1	0.5
Cushing’s syndrome	1	0.50	Steroid-induced psychosis	1	0.5
Myoma uterus	1	0.50	Chloroquine-drug allergy	1	0.5
Epilepsy	3	2	Diarrhea	2	1.0
Liver Hemangioma	4	2	Leucopenia	2	1.0
Hyperlipidemia	3	1.50	Maculopathy	2	1.0
Schizophrenia	2	1	Ophthalmopathy	2	1.0
Glaucoma	2	1	Osteonecrosis	1	0.5

### Treatment-related characteristics of lupus nephritis patients

The most common drugs during the induction phase were mycophenolate mofetil (MMF) with prednisolone 99 (49.5%) and cyclophosphamide (CYC) with prednisolone 62 (31%). Regarding maintenance therapy, the most frequently prescribed drugs were MMF with prednisolone 120 (60%), and prednisolone alone 42 (21%). Only 24.5% of patients received three days of pulse steroid therapy which is comprised of either methylprednisolone or prednisolone. The dose of prednisolone used in pulse steroid therapy in this study is in the range of 80–110 mg per day. The total daily dose of prednisolone was divided into morning and evening doses for three days when used for pulse steroid therapy. The drug most widely used for pulse steroid therapy in lupus nephritis patients is methylprednisolone; however, given its easy availability and cost, high-dose prednisolone has been often used in Ethiopia for pulse steroid to decrease the severity of diseases. Patients with an overall complete plus partial response to immunosuppressive therapy during the induction therapy were 185 (92.5%). From this, LN patients who achieved partial response (57%) were higher than those who achieved complete response (35.5%). The median duration of induction and maintenance therapy was 6 (6–8) months and 20 (18–25) months, respectively. Prophylactic cotrimoxazole was used in approximately half of the patients. In this study, regimen changes in both induction and maintenance therapy for LN were observed in 17 and 18 LN patients, respectively. The most common reason for the regimen change was non-responsiveness to immunosuppressive therapy, with only two patients on metformin due to medication error (Table 4).

**Table 4:** Treatment-related characteristics of LN patients on follow-up at the renal clinic of TASH and SPHMMC from January 1, 2016 to January 1, 2021 (n = 200).

Variables	Category	N	Treatment outcome			*p* value
			CR n (%)	PR n (%)	NR n (%)	
Pulse steroid	Yes	49	38 (19)	6 (3)	5 (2.5)	0.169
Pulse steroid given	Methylprednisolone	33	24 (12)	5 (2.5)	4 (2)	0.328
	Prednisolone	16	14 (7)	1 (0.5)	1 (0.5)	
IV MP dose/day	500 mg	21	14 (7)	3 (1.5)	4 (2)	0.582
	1000 mg	12	10 (8)	2 (1)	0 (0)	
Pred dose/day	> 80 mg/day	16	14 (7)	1 (0.5)	1 (0.5)	0.281
Induction therapy	Prednisolone	39	27 (13.5)	8 (4)	4 (2)	0.080
	MMF with Pred	99	63 (31.5)	23 (11.5)	13 (6.5)	
	CYC with Pred	62	43 (21.5)	5 (2.5)	14 (7)	
Induction MMF	2000 mg/day	87	57 (28.5)	20 (10)	10 (5)	0.134
	1000 mg/day	12	5 (2.5)	4 (2)	3 (1.5)	
Induction CYC	< 750 mg/month	25	14 (7)	3 (1.5)	8 (4)	0.019
	≥ 750 mg/month	37	29 (14.5)	2 (1)	6 (3)	
Pred starting dose	> 50 mg/day	148	97 (48.5)	26 (13)	25 (12.5)	0.654
	≤ 50 mg/day	52	30 (18)	10 (5)	6 (3)	
Induction duration	Above 6 month	60	35 (17.5)	12 (6)	13 (6.5)	0.207
	6 month	140	98 (49)	24 (12)	18 (9)	
Response to induction therapy	Complete response	71	65 (32.5)	3 (1.5)	3 (1.5)	0.000
	Partial response	114	65 (32.5)	32 (16.0)	17 (8.5)	
	Non-response	15	3 (1.5)	1 (0.5)	11 (5.5)	
Maintenance therapy	MMF with Pred	120	83 (41.5)	22 (11)	15 (7.5)	0.132
	CYC with Pred	6	3 (1.5)	1 (0.5)	2 (1)	
	AZA with Pred	32	19 (9.5)	4 (2)	9 (4.5)	
	Prednisolone alone	42	28 (14)	9 (4.5)	5 (2.5)	
MMF maintenance	2000 mg/day	7	3 (1.5)	3 (1.5)	1 (0.50	0.434
	≤ 1000 mg/day	114	80 (40)	19 (9.5)	15 (7.50	
CYC maintenance	≤ 750 mg/3 month	6	3 (1.5)	1 (0.5)	2 (1)	0.466
AZA maintenance	150 mg/day	2	1 (0.5)	0	1 (0.5)	0.375
	100 mg/day	29	18 (9)	4 (2)	7 (3.5)	
Pred maintenance	≤ 20 mg/day	177	119 (59.5)	34 (17)	24 (12)	0.078
	> 20 mg/day	23	14 (7)	2 (1)	7 (3.5)	
Maintenance duration	> 24 months	73	54 (27)	15 (5.5)	8 (4)	0.128
	≤ 24 months	127	79 (39.5)	25 (12.5)	23 (11.5)	
Remission time	≤ 3 months	65	48 (24)	8 (4)	9 (4.5)	0.241
	> 3 months	135	85 (42.5)	28 (14)	22 (11)	
Total follow up	≥ 24 months	176	120 (60)	32 (16)	24 (12)	0.140
	< 24 months	24	13 (6.5)	4 (2)	7 (3.5)	
TMT-SMT	Yes	102	77 (38.5)	17 (8.5)	8 (4)	0.005

*IV* intravenous, *MP* methylprednisolone, *MMF* mycophenolate mofetil, *CYC* cyclophosphamide, *AZA* azathioprine, *TMT-SMT* trimethoprim-sulfamethoxazole.

### Factors associated with treatment outcome of lupus nephritis patients

According to the multinomial logistic regression model; LN patients with moderate SLE disease activity index (AOR = 6.25, 95% CI 1.49–26.10), took pulse steroid (AOR = 5.68, 95% CI 0.99–32.3), started induction therapy with prednisolone only (AOR = 85.79, 95% CI 1.16–635.0) or CYC with prednisolone (AOR = 85.79, 95% CI 1.16–635.0), completed induction therapy at 6 months (AOR = 16.35, 95% CI 1.50–180.75), achieved complete response (AOR = 200.8, 95% CI 16.5–2437.6) or partial response (AOR = 20.26, 95% CI 1.96–209.4) after 6 months of induction therapy, placed on maintenance therapy of MMF with prednisolone (AOR = 69.15, 95% CI 2.818–1442.0), or AZA with prednisolone (AOR = 72.1, 95% CI 1.16–447.0), received cotrimoxazole prophylaxis (AOR = 7.48, 95% CI 1.76–31.87), and had baseline leucopenia (AOR = 14.2, 95% CI 1.04–201.3) were more likely to achieve a complete response at their last follow-up visit as compared to the non-responders (Table 5).

**Table 5:** Multinomial logistic regression analysis of factors associated with treatment outcome among LN patients on follow-up at the renal clinic of TASH and SPHMMC from January 1, 2016 to January 1, 2021 (n = 200).

Variable category	LN treatment outcome							
	CR versus NR				PR versus NR			
	COR	AOR	*P* value	95% CI	COR	AOR	*P* value	95% CI
Age								
15–30 years	0.99	0.88	0.865	0.22–3.56	1.87	3.42	0.141	0.66–17.5
> 30 years	1	0.1	1	1.0	1	1.0	0.1	0.1
Diagnosis								
Without biopsy	0.48	0.42	0.307	0.08–2.20	0.45	0.25	0.137	0.04–1.55
With biopsy	1	0.1	1	1.0	1	0.1	1	1.0
Residence								
Outside AA	1.87	2.45	0.240	0.55–10.90	1.86	2.07	0.380	0.41–10.4
From AA	1	0.1	1	1.0	1	0.1	1	1.0
SLE duration								
< 5 years	0.64	0.25	0.121	0.04–1.45	0.52	0.19	0.094	0.03–1.32
> 5 years	1	0.1	1	1.0	1	0.1	1	1.0
SLE Dx severity								
Moderate	2.40	6.25	**0.012**	1.49–26.10	1.66	2.05	0.383	0.41–10.3
Severe	1	0.1	1	1.0	1	0.1	1	1.0
Comorbidity (yes)	0.54	0.67	0.547	0.18–2.45	0.34	0.21	**0.038**	0.05–0.92
Baseline Scr								
≤ 1.4 mg/dl	1.71	3.34	0.141	0.67–16.50	1.76	1.92	0.466	0.33–11.2
> 1.4 mg/dl	1	0.1	1	1.0	1	0.1	1	1.0
Baseline 24 urine								
≤ 3 g/day	1.32	2.01	0.365	0.44–9.12	0.43	0.45	0.320	0.09–2.19
> 3 g/day	1	0.1	1	1.0	1	0.1	1	1.0
Pulse steroid (yes)	2.32	5.68	**0.048**	0.99–32.30	1.05	1.43	0.730	0.17–10.9
Induction therapy								
Prednisolone	2.61	85.79	**0.043**	1.16–635.0	5.64	8.76	0.335	0.11–72.7
MMF with pred	1.49	1.51	0.655	0.25–9.13	4.94	17.55	**0.015**	1.76–174
CYC with pred	1	0.1	1	1.0	1	0.1	1	1.0
Induction duration								
6 month	4.50	16.35	**0.023**	1.50–180.7	3.15	19.47	**0.024**	1.5–258.8
Above 6 month	1	0.1	1	1.0	1	0.1	1	1.0
Induction response								
Complete response	72.79	200.8	**0.000**	16.5–2437	10.9	16.85	0.115	0.5–564.0
Partial response	13.5	20.26	**0.012**	1.96–209.0	20.7	32.63	**0.028**	1.4–736.0
Non-response	1	0.1	1	1.0	1	0.1	1	1.0
Maintenance TT								
MMF with pred	1.01	69.2	**0.036**	2.8–1442.0	0.83	1.16	0.836	0.02–15.0
CYC with pred	0.23	15.9	0.325	0.06–39.10	0.28	21.05	0.260	0.02–30.0
AZA with pred	0.08	72.1	**0.042**	1.16–447.0	0.23	2.66	0.667	0.02–51.0
Prednisolone	1	0.1	1	1.0	1	0.1	1	1.0
Maintenance dur								
> 24 months	2.36	2.61	0.178	0.65–10.48	1.443	0.69	0.647	0.14–4.0
≤ 24 months	1	0.1	1	1.0	1	0.1	1	1.0
Maintenance pred								
≤ 20 mg/day	2.45	1.71	0.549	0.29–9.96	4.91	7.09	0.096	0.70–71.0
> 20 mg/day	1	0.1	1	1.0	1	0.1	1	1.0
Remission time								
≤ 3 months	1.59	1.48	0.593	0.35–6.19	0.78	0.54	0.461	0.10–2.80
> 3 months	1	0.1	1	1.0	1	0.1	1	1.0
Total follow up								
≥ 24 months	2.97	2.33	0.392	0.34–16.09	2.223	1.93	0.555	0.20–170
< 24 months	1	0.1	1	1.0	1	0.1	1	1.0
TMT-SMT (yes)	4.52	7.48	**0.006**	1.76–31.87	2.65	2.69	0.241	0.50–140
Admission (yes)	0.26	0.32	0.091	0.09–1.20	0.39	0.31	0.129	0.10–1.40
Leucopenia (yes)	9.71	14.2	**0.047**	1.04–201.3	4.87	5.89	0.221	0.30–10.0

*Dx* disease, *CI* confidence interval, *AOR* adjusted odds ratio, *COR* crude odds ratio, *AA* Addis Ababa, *SLE* systemic lupus erythematous, *dur* duration, *MMF* mycophenolate mofetil, *CYC* cyclophosphamide, *TT* treatment, *Scr* serum creatinine, *Pred* prednisolone, *AZA* azathioprine, *TMT-SMT* trimethoprim sulfamethoxazole.

Significant values are in bold.

On the other hand, patients who started MMF with prednisolone induction therapy (AOR = 17.55, 95% CI 1.76–174.0), completed induction therapy at 6 months (AOR = 19.47, 95% CI 1.46–258.8), and achieved partial response after 6 months of induction therapy (AOR = 32.63, 95% CI 1.45–736.0) were more likely to attain a partial response at their last follow-up as compared to the non-responders. Conversely, patients with comorbid conditions (AOR = 0.21, 95% CI 0.05–0.92) were less likely to achieve partial response as compared to the non-responders (Table 5).

### Factors associated with lupus nephritis patients' survival

During bivariate Cox regression analysis, baseline serum creatinine ≥ 1.4 mg/dl (*P* = 0.034) and increased white blood cell (*P* = 0.016) were associated with an increased risk of non-response in LN patients on follow-up, whereas complete response (*P* = 0.000) and partial response (*P* = 0.000) to induction therapy were associated with decreased risk of non-response.

However, during multivariate Cox regression analysis, only response to induction therapy was significantly associated with a reduction in non-response. Accordingly, patients with complete responses at initial treatment were found to have an approximately 93.1% decreased risk of non-response as compared with patients with non-response at initial therapy. Similarly, patients with a partial response at initial treatment were found to have nearly 75.1% decreased risk of non-response as compared with patients with non-response at initial therapy (Table 6).

**Table 6:** Cox-regression survival analysis of adult LN patients on follow-up at TASH and SPHMMC renal clinics between January 1, 2016 and January 1, 2021 (n = 200).

Factors	CHR (95% CI)	*P* value	AHR (95% CI)	*P* value
Age in years				
15–30	1.062 (0.58–1.95)	0.845		
> 30	1			
SLE Dx severity				
Moderate	1.54 (0.83–2.86)	0.173	0.698 (0.34–1.38)	0.300
Severe	1		1	
Baseline serum Cr				
≤ 1.4 mg/dl	0.484 (0.25–0.95)	**0.034**	0.585 (0.27–1.27)	0.175
> 1.4 mg/dl	1		1	
24 h urine protein				
≤ 3 g/day	1.378 (0.75–2.54)	0.305		
> 3 g/day	1			
Baseline Hgb	0.919 (0.82–1.02)	0.123	0.941 (0.83–1.07)	0.340
Baseline WBC	1.093 (1.02–1.17)	**0.016**	1.009 (0.93–1.09)	0.878
Admission (yes)	1.682 (0.86–3.29)	0.130	0.907 (0.47–1.74)	0.769
Induction therapy				
Pred only	0.648 (0.29–1.46)	0.295	1.576 (0.20–12.05)	0.669
Pred with MMF	0.530 (0.28–1.02)	0.056	0.637 (0.32–1.29)	0.209
Pred with CYC	1			
Induction response				
Complete response	0.078 (0.03–0.22)	**0.000**	0.087 (0.07–0.29)	**0.000**
Partial response	0.234 (0.12–0.45)	**0.000**	0.249 (0.11–0.57)	**0.000**
Non-response	1		1	
Maintenance therapy				
MMF with pred	1.225 (0.55–2.72)	0.446	1.232 (0.14–10.92)	0.851
CYC with pred	2.902 (0.78–10.81)	0.112	2.232 (0.26–19.05)	0.463
AZA with pred	1.42 (0.56–3.63)	0.464	0.740 (0.07–7.73)	0.802
Pred only	1		1	
TMT-SMT (yes)	0.622 (0.33–1.17)	0.124	0.702 (0.36–1.37)	0.298

*CHR* crude hazard ratio, *AHR* adjusted hazard ratio, *Cr* creatinine, *Hgb* hemoglobin, *WBC* white blood cell, *Pred* prednisolone, *MMF* mycophenolate mofetil, *CYC* cyclophosphamide, *AZA* azathioprine, *TMT-SMT* Trimethoprim-sulfamethoxazole, *Dx* disease.

Significant values are in bold.

**Figure 1:**
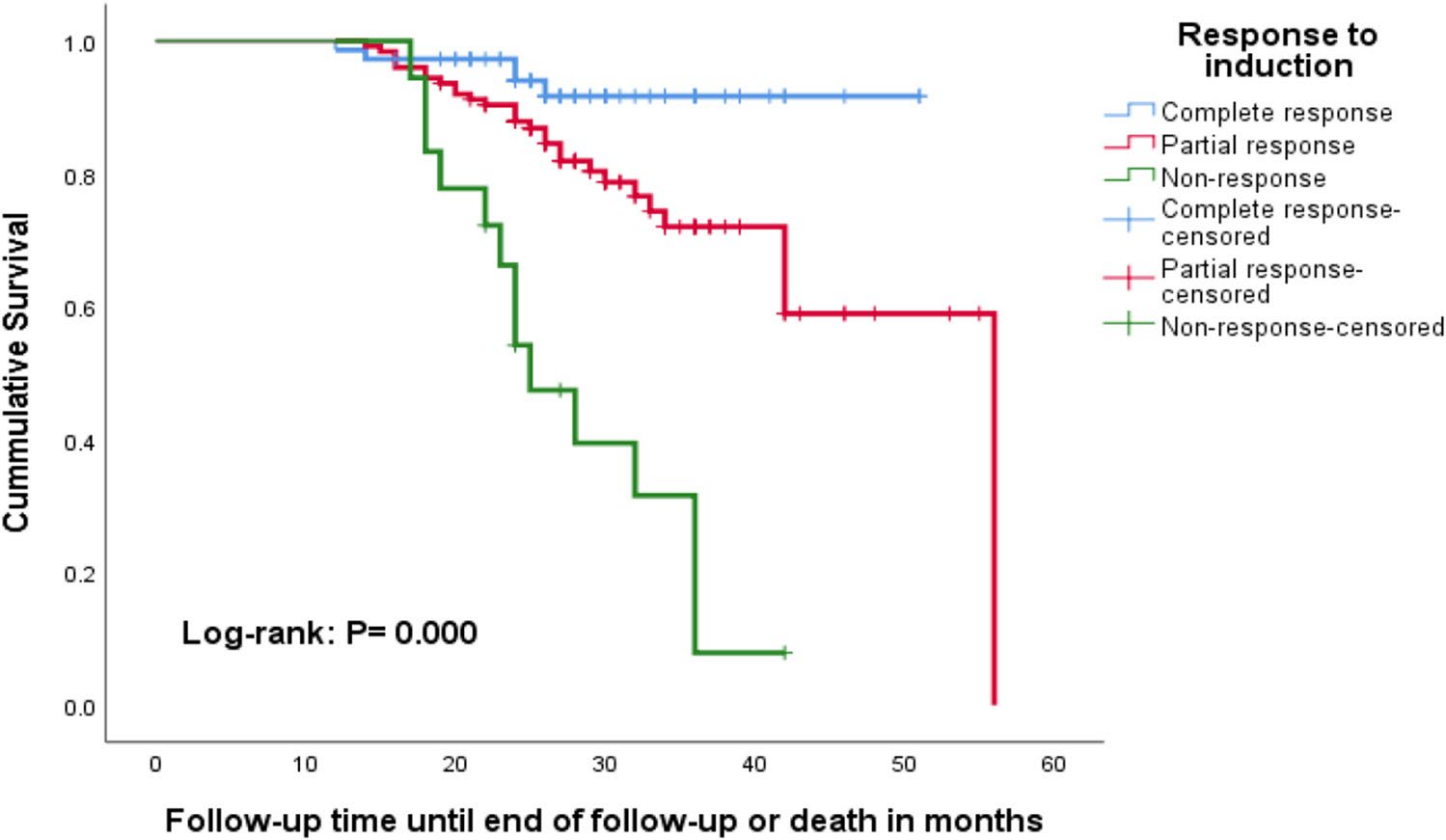
Kaplan–Meier survival analysis of LN patients on follow-up stratified by response to induction therapy.

To support the Cox regression analysis finding, Kaplan–Meier survival analysis was performed by considering the period from treatment initiation to end of follow-up or death as the time frame. Based on Kaplan–Meier analysis, complete and partial responses to initial therapy were independent factors associated with decreased risk of non-response. The survival of LN patients with a complete response to initial therapy was greater than the survival of patients with a non-response to initial therapy (*P* = 0.000 by log-rank test). Similarly, the survival of LN patients with partial response during initial therapy was greater than the survival of those patients with non-response at initial therapy (*P* = 0.000 by log-rank test) (Fig. 1).

The 24-month survival rate for LN patients who achieved CR after induction therapy and those who achieved PR were 94.02% and 87.86%, respectively while non-responders had a survival rate of 54.17% at 24 months. Patient survivals at the end of follow-up were 91.67%, 58.93%, and 7.9% for patients with complete, partial, and non-response to initial therapy, respectively. The overall median survival time for LN patients with partial response and non-response at initial therapy was 56 months and 25 months, respectively (Fig. 1).

## Discussion

This study is one of the few that assesses the treatment outcome, survival status, and associated factors of LN patients in the sub-Saharan Africa (SSA) contexts, particularly in Ethiopia. It is also the pioneer study to profoundly examine multiple factors associated with LN treatment outcome, and survival status by using a dual-center setting containing a relatively larger sample.

In this study, remission rate to treatment (complete plus partial response) is around 92.5% at the end of induction therapy with immunosuppressant drugs. This finding is in line with a study done in China in which nearly 90% of patients achieved remission after induction therapy consisting of prednisolone with either MMF or CYC^21^. However, it was relatively higher than the studies done in Ibn Sina University Hospital of Morocco, and Aristide Le Dante University Hospital of Senegal, in which 66% and 57.14% of patients achieved remission, respectively^22,23^. The discrepancy could be attributed to the differences in the selection of treatment regimen (MMF with prednisolone vs CYC alone or AZA alone), male-to-female sex ratio (0.09 vs 0.23 and 0.09 vs 0.128), and sample size (200 vs 114 and 200 vs 93). For instance, CYC alone was utilized in almost all cases of LN from Morocco due to low cost, whereas MMF was mainly utilized in our study by combining with prednisolone. Moreover, the current finding was relatively lower than a study done in India^24^, which reported 94.1% of remission (complete plus partial). The variation could also be attributed to differences in the inclusion criteria (age; 15–60 years vs < 18 years), minimum follow-up time (1 year vs 5 years), genetic difference, and definition of response.

At the last visit of follow-up, about 84.5% LN patients achieved remission (complete plus partial response). Out of these, 66.5% of them had a complete response [CR] while 18% had a partial response [PR]. A similar observation was noted in a study done in Italy in which 82.8% of LN patients achieved remission at the last follow-up^25^. However, this finding was relatively higher than the finding of a study done in Libya which reported a complete, partial, and non-response in 64.5%, 13.3%, and 22.3%, respectively^26^. This incongruity might be ascribed to the variation in the definition of response, magnitude of comorbidity like hypertension (38.5% vs 89.5%), choice of treatment protocol followed (ACR vs EULAR), sample size (200 vs 76), and use of adjuvant chloroquine (100% vs 0%)^27^. The current finding was relatively lower compared to a previous study done in Russia^28^, where overall remission (CR plus PR) was reported to be around 95.7%. Likewise, this is mostly due to differences in length of follow-up (5 years vs 23 years), genetics, socioeconomic status, and type of regimen selected for maintenance therapy.

Among the baseline laboratory tests, lupus serology (positive ANA and anti-dsDNA), serum creatinine, and 24-h urine protein were not significantly associated with either CR or PR in this study. These findings were consistent with a study done in Turkey^8^. In this study, the male gender had no statistically significant contribution to non-response, even though several studies^29–31^ identified it as a risk factor. This could be due to the small number of male participants (N = 17) in our study, and genetic or hormonal differences. Similar to a study done in Senegal, the presence of leucopenia at baseline was significantly associated with non-response^23^. However, this finding is not consistent with many previous studies^22,26,32,33^. This could be partly due to variations in laboratory test control, sample size, and the presence of infection.

In the present study, LN patients who had a severe SLE disease activity index were less likely to achieve a CR than NR as compared with patients with a moderate disease activity index. This real-world finding conforms with studies conducted in Taiwan tertiary referral center, West China Hospital of Sichuan University, and India^33–35^ but is incongruent with a study conducted in Egypt^36^. The incongruity might be ascribed to the higher proportion of patients receiving three days high dose pulse steroid therapy (24.5% vs 100%) and the smaller sample size (85 vs 200).

Our study differs from other studies^23,26^ concerning the impact of pulse steroid therapy on treatment outcomes. In our study, the use of pulse steroid therapy was significantly associated with CR though only a few patients (24.5%) received it. This finding is in agreement with studies conducted in Japan and Senegal^37,38^. In contrast, a study conducted in Pakistan found that pulse steroids had no statistically significant effect on LN treatment outcomes. This dispute is likely due to nearly 90% of patients receiving only oral corticosteroids after pulse steroid therapy in Pakistan while either CYC or MMF was initiated in our case^39^.

Our findings revealed that the presence of comorbidity is significantly associated with LN non-response. This finding was supported by a prospective study done in Egypt^36^, in which 81.1% of non-responders had comorbidity at the initial. Conversely, patients with comorbidity were not significantly associated with a reduction in PR in two previous studies^23,33^. This discrepancy could be attributed to the merging of complete and partial responses.

In this study, partial response is more likely to occur in patients taking MMF with prednisolone than in patients taking CYC with prednisolone during induction therapy. This finding was similar to a study done in the United States of America in which most patients were black African^40^. This is because MMF is more effective than CYC in black African patients. Prolonged duration of induction therapy (more than 6 months) favors NR when compared with 6-month therapy, possibly because of non-adherence, drug toxicity, and medication error. Using either MMF or AZA as maintenance therapy is likely associated with a statistically significant CR compared with prednisolone alone. This finding is consistent with various international guidelines^4,13^.

LN patients who did not respond completely or partially to induction therapy after 6 to 12 months were significantly more likely to have non-response after long-term follow-up than their counterparts. Related studies have found that patients who achieve a partial or complete response after induction therapy had a greater CR than non-responders after long-term follow-up^28,41^. In this study, LN patients taking prophylactic cotrimoxazole achieved a statistically significant CR better than NR as compared to patients not taking prophylaxis. This is due to the benefit of cotrimoxazole in preventing infections caused by the immunosuppressant drugs used in LN^42^. In various studies, the intention of using cotrimoxazole was as a prophylaxis for opportunistic infections, but in the current study, we come up with a new hypothesis of using cotrimoxazole to increase the complete response in patients due to the reduction of antibodies related to B-cell productions secondary to bone marrow suppressions.

Survival of patients attaining complete response at the last follow-up visit was more than 90%. This finding was in keeping with two previous studies^16,43^. In this study, 14.4% of patients were non-responders at the end of the follow-up visit. This finding is lower than previous studies done in Italy^25^ (17.2%) and Chicago^16^ (32.0%) but higher than a study done in Egypt^36^ (12.9%). The incongruity could be ascribed to the differences in sample size, class of LN, the definition of remission, diagnosis by biopsy, age, and genetics.

Our survival analysis revealed that patients attaining complete response after induction therapy were shown to have an excellent prognosis. The current study finding unveiled that those patients achieving complete and partial responses to induction therapy decreased the risk of non-responders by 93.1% and 75.1%, respectively. So, non-response to induction therapy negatively influenced patient survival. This finding was consistent with three previous studies^16,25,28^.

Our study had the strength of being a dual center with a relatively higher number of patients compared with most other studies. Moreover, most of our patients have stayed on follow-up for more than 24 months and treatment outcomes were evaluated individually. Nevertheless, our study may have been limited by its retrospective study design and variation of treatment regimens used to treat LN which resulted in variations in treatment outcomes. Besides this, around 28% of patients were diagnosed with LN without renal biopsy in our study even though renal biopsy was the gold standard diagnostic method for LN. Furthermore, the reasons for those patients who have been lost to follow-up have not been captured. Lastly, due to the high cost and interrupted supply of immunosuppressive medications, some LN patients were shifted from one regimen to another which may have impacted treatment outcomes.

## Conclusion

Treatment of LN sounds better in terms of treatment outcome and patient survival in our study setting. More than three-fourths of LN patients respond to drug therapy. Our findings revealed that the use of pulse steroids, complete response or partial response at the induction therapy, administration of MMF with prednisolone, AZA with prednisolone, and use of prophylactic cotrimoxazole were significantly associated with a favorable treatment outcome whereas the presence of leucopenia, comorbidity, induction duration more than six months, non-response to induction therapy and severe disease index were significantly associated with lower probability of complete or partial response (Supplementary Information S1).

### Supplementary Information

Supplementary Information.

## Data Availability

The datasets used and/or analyzed during the current study are available from the corresponding author upon reasonable request.

## Abbreviations

ANA: Antinuclear antibodies
AOR: Adjusted odds ratio
AZA: Azathioprine
COR: Crude odds ratio
CR: Complete response
CYC: Cyclophosphamide
ESRD: End stage renal disease
LN: Lupus nephritis
NR: Non-response
PR: Partial response
TASH: Tikur Anbessa specialized hospital
SLE: Systemic lupus erythematous
SPHMMC: Saint Paul Hospital Millennium Medical College
