# Supplementary material for: Treatment outcome and survival status among adult patients treated for lupus nephritis in selected tertiary hospitals of Ethiopia
Source: Sci Rep. 2024 Mar 7;14:5603. doi: 10.1038/s41598-024-56317-6 (PMC10920682; doi:10.1038/s41598-024-56317-6)
Supplement: Supplementary file 1 — Supplementary Information. [file 41598_2024_56317_MOESM1_ESM.pdf]

Treatment Outcome and Survival Status Among Adult Patients Treated for Lupus Nephritis in Selected Tertiary Hospitals of Ethiopia

Oumer Aliyi[1], Berhanu Worku[2], Minimize Hassen[3], Oumer Sada Muhammed[4]*

ANNEXES

ANNEX 1: Check List of Data Collection Format

Demographic Patient Information

1. Card number___________
2. Sex: a) Male   b) Female
3. Age in years______
4. Region__________________
5. Diagnosis category ______________________
6. Duration of SLE before LN onset__________________

Baseline Patient Clinical Characteristics and Laboratory Value

1. Class of lupus nephritis by biopsy result
   a) Class II b) Class III c) Class IV d) Class V e) mixed f) Not recorded
2. Systemic lupus erythematosus disease activity index score (SLEDAI)
   a) Mild b) Moderate c) Severe
3. Baseline laboratory value at treatment initiation and during follow up

		Baseline	At 3month	At 6 months	At 12 months	At last follow-up
Scr						
24hrs protein						
ANA						
dsDNA						
Hgb						
Total WBC						
PLT						
UA	Leukocyte					
	Protein					
	RBC					
	WBC					

Drug Therapy

4. Initial treatment regimen selected for treatment

   1) MMF 2) Cyclophosphamide 3) Prednisolone 4) Prednisolone + MMF

   5) Prednisolone + Cyclophosphamide 6) Chloroquine 7) Cotrimoxazole

5. Starting dose: a) MMF__________ b) Cyclophosphamide____________ c) Prednisolone_____
6. Pulse steroid given a) Yes b) No
7. Dose and duration of pulse steroid:

   ____

Induction and Maintenance Therapy During Follow-up

A) Treatment regimen selected for induction therapy: a) MMF_________ b) CYC__________

c) Prednisolone_________ d) chloroquine_________ e) Cotrimoxazole __________

1. Duration of induction therapy_____________________________________
2. Drug tapered during induction___________________________________
3. Regimen modification during induction therapy: a) MMF to Cyclophosphamide b) Cyclophosphamide to MMF c) Dose reduction d) No-change
4. Reason for dose modification______________________________
5. Response to induction therapy a) Complete response b) Partial response c) Non-response

B) Treatment regimen selected for maintenance therapy:

a) MMF___________ b) CYC__________ c) Prednisolone_________ d) Azathioprine

e) Chloroquine_________ f) Cotrimoxazole _____________

1. Duration of maintenance therapy __________________________________
2. Drug tapered during maintenance__________________________
3. Switching of initial maintenance therapy to other drugs A) Yes b) No
4. If yes switched to which drugs____________________________________
5. Reason for switching____
6. Frequency of follow up interval__________
7. Total follow up period______________________
8. Time to start remission (in month) ---------------------
9. Admission: 1) Yes 2) No

10. If admitted, cause of admission:

____

Patient Condition at the End of Follow-up

1. Response 1) complete response 2) Partial response 3) No-response

   If No- response: a) Reinduction by the same regimen b) Alternative drug considered
2. ESRD
3. Death
4. Loss to follow up
5. Relapse: 1) Yes 2) No

Comorbidity

Comorbidity: 1) Yes 2) No

Comorbidity developed during follow-up _____________________________

Drug given for comorbidity management_____________________________

ANNEX 2: Treatment Outcome Status Definition by mALMS Criteria

Ordinary remission criteria	mALMS criteria
**Complete response**	Returning to normal serum creatinine (Serum creatinine ≤ 1.4mg/dl) **AND** urine protein ≤ 0.5 g/day (UPCR ≤ 0.5- or 24-hours urine protein (g/day) ≤ 0.5g/day
**Partial response**	Returning to normal serum creatinine (Serum creatinine ≤ 1.4mg/dl) **OR** urine protein ≤ 0.5 g/day **(**UPCR≤ 0.5- or 24-hours urine protein (g/day) ≤ 0.5 g/day
**No-response**	Not fulfilling the criteria of complete or partial response

GFR, glomerular filtration rate; JHU, John Hopkins University; mALMS, modified Aspreva Lupus Management Study; UPCR - urine protein to creatinine ratio

ANNEX 3: American College of Rheumatology 1982 Revised Criteria (Tan et al., 1982)

Criteria	Definition
1. Malar "butterfly" rash	Fixed erythema, flat or raised, over the malar eminences, tending to spare the nasolabial folds
2. Discoid rash	Erythematosus raised patches with adherent keratotic scaling and follicular plugging; atrophic scarring may occur in older lesions.
3. Photosensitivity	Skin rash as a result of unusual reaction to sunlight, by patient history or physician observation.
4. Oral ulcers	Oral or nasopharyngeal ulceration usually painless observed by physician.
5. Arthritis	Non-erosive arthritis involving 2 or more peripheral joints characterized by tenderness, swelling, or effusion.
6. Serositis	a) Pleuritis (convincing history or pleuritic pain or rub heard by physician or evidence of pleural effusion), OR<br>b) Pericarditis (documented by ECG, rub, or evidence or pericardial effusion).
7. Renal disorder	a) Persistent proteinuria (> 0.5 grams/day or > 3 + if quantitation not performed) OR<br>b) Cellular casts (maybe red cell, hemoglobin, granular, tubular, or mixed).
8. Neurologic disorders	a) Seizures (in the absence of offending drugs or known metabolic derangements like uremia, ketoacidosis, or electrolyte imbalance) OR<br>b) Psychosis (in the absence of offending drugs or known metabolic derangements; i.e., uremia, ketoacidosis, or electrolyte imbalance.
9. Hematologic disorders	a. Hemolytic anemia (with reticulocytosis) OR<br>b. Leukopenia (< 4000/mm3 total on 2 or more occasions),OR<br>c. Lymphopenia (< 100,000/mm3 in the absence of offending drugs).
10. Immunologic disorders	a. Anti-DNA (antibody to native DNA in abnormal titer), OR<br>b. Anti-Sm (presence of antibody to Sm nuclear antigen), OR<br>c. Positive-finding of antiphospholipid antibodies based on 1) an abnormal serum level of IgG or IgM anticardiolipin antibodies, 2) a positive test result for lupus anticoagulant using a standard method, or 3) a false-positive serologic test for syphilis known to be positive for at least 6 months and confirmed by Treponema pallidum immobilization (TPI) or fluorescent treponemal antibody (FTA) absorption test.
11. Antinuclear antibody (ANA)	Abnormal titer of ANA by immunofluorescence or an equivalent assay at any point in time and in the absence of drugs known to be associated with "drug-induced lupus" syndrome.

* A person shall be said to have systemic lupus erythematous if any 4 or more of the 11 criteria are present, serially or simultaneously, during any interval or observation.

ANNEX 4: Systemic Lupus Erythematous Disease Activity Index (SLEDAI-2K) (Gladman *et al*., 2002)

Physician Global Assessment ____

0-None, 1-Mild, 2-Moderate, 3-Severe

Weight	Description	Definition
8	Seizure	Recent onset. Exclude metabolic, infectious or drug cause
8	Psychosis	Altered ability to function in normal activity due to severe disturbance in the perception of reality. It includes hallucinations, incoherence, marked loose association, marked illogical thinking, bizarre, disorganized or catatonic behavior. Excluded uremia and drug cause
8	Organic Brain Syndrome	Altered mental function with impaired orientation, memory or other intelligent function, with rapid onset fluctuating clinical features. Includes clouding of consciousness with reduced capacity to focus and inability to sustain attention to environment plus at least two of the following:<br>Perceptual disturbance, incoherent speech, insomnia or daytime drowsiness or increases or decreased psychomotor activity. Exclude metabolic, infectious or drug cause.
8	Visual disturbance	Retinal changes like cystoids bodies, retinal hemorrhage, serious exudate or hemorrhages in the choroids and optic neuritis. Exclude hypertension, infection and drug causes.
8	Cranial nerve disorders	New onset of sensory or motor neuropathy involving cranial nerves.
8	Lupus headache	Severe persistent headache that might be migraineous, must be non-responsive to narcotic analgesia.
8	Cardiovascular accident	New onset of cerebrovascular accident. Exclude arteriosclerosis
8	Vasculitis	Ulceration, gangrene, tender finger nodule, periungual, infarction, splinter hemorrhages and biopsy proof of vasculitis
4	Arthritis	More than two joint with pain and signs of inflammation (tenderness, swelling and effusion)
4	Myositis	Proximal muscle aching/weakness that associated with elevated creatinine phosphokinase or biopsy showing myositis
4	Urinary cast	Granular or red blood cell casts

<table>
<tr><td>4</td><td>Hematuria</td><td colspan="3">Greater than 5 red blood cells/high power field. Exclude stone, infection, or other cause</td></tr>
<tr><td>4</td><td>Proteinuria</td><td colspan="3">Greater than 0.5 g/day. New onset or recent increase of more than 0.5g/day</td></tr>
<tr><td>4</td><td>Pyuria</td><td colspan="3">Greater than 5 white blood cells/high power field. Exclude infection</td></tr>
<tr><td>2</td><td>New rash</td><td colspan="3">New onset or recurrence of inflammatory-type rash.</td></tr>
<tr><td>2</td><td>Alopecia</td><td colspan="3">New onset or recurrence of abnormal, patchy, or diffuse loss of hair</td></tr>
<tr><td>2</td><td>Mucosal ulcers</td><td colspan="3">New onset or recurrence mucosa or nasal ulcers</td></tr>
<tr><td>2</td><td>Pleurisy</td><td colspan="3">Pleuritic chest pain with pleural rub or effusion or pleural thickening</td></tr>
<tr><td>2</td><td>Pericarditis</td><td colspan="3">Pericardial pain with at least one of the following: rub, effusion, or electrocardiogram confirmation.</td></tr>
<tr><td>2</td><td>Low complement</td><td colspan="3">Decreased C3 or C4 to below the lower limit of normal test laboratory</td></tr>
<tr><td>2</td><td>Increased DNA binding</td><td colspan="3">Greater than 25% binding by farr assay or above normal range for testing laboratory</td></tr>
<tr><td>1</td><td>Fever</td><td colspan="3">Greater than 38 ˚C. Exclude infectious cause</td></tr>
<tr><td>1</td><td>Thrombocytopenia</td><td colspan="3">Less than 100,000 cell/mm^3</td></tr>
<tr><td>1</td><td>Leucopenia</td><td colspan="3"><300,000 cell/mm3</td></tr>
<tr><td>105</td><td colspan="4">Total SLEDAI score</td></tr>
<tr><td rowspan="2"></td><td colspan="4">SLEDAI-2K reference range interpretation</td></tr>
<tr><td>No flare</td><td>Mild</td><td>Moderate flare</td><td>Severe flare</td></tr>
<tr><td>Total SLEDAI Score</td><td>0</td><td>1-3</td><td>4-12</td><td>>12</td></tr>
</table>
